# Six Cases of Hairpin in the Bladder

**Published:** 1912-10

**Authors:** 


Six Cases of Hairpin in the Bladder. Adame, *Riv. di Med. y Cirujia Pratic.*, June 28, 1911. Five cases were in young girls one in a multipara. Three were subjected to abdominal section, one to vaginal, two to extraction by the urethra, all with perfect results. In addition, he notes 8 cases of calculus of the bladder not due to foreign bodies, equally successfully treated.

